# Age and the Neural Network of Personal Familiarity

**DOI:** 10.1371/journal.pone.0015790

**Published:** 2010-12-22

**Authors:** Markus Donix, Katja Petrowski, Luisa Jurjanz, Thomas Huebner, Ulf Herold, Damaris Baeumler, Eva C. Amanatidis, Katrin Poettrich, Michael N. Smolka, Vjera A. Holthoff

**Affiliations:** 1 Department of Psychiatry and Psychotherapy, University Clinic Carl Gustav Carus, Technische Universität Dresden, Dresden, Germany; 2 Department of Psychotherapy and Psychosomatic Medicine, University Clinic Carl Gustav Carus, Technische Universität Dresden, Dresden, Germany; 3 Neuroimaging Center, Department of Psychology, Technische Universität Dresden, Dresden, Germany; 4 DZNE, German Center for Neurodegenerative Diseases, Dresden, Germany; The University of Western Ontario, Canada

## Abstract

**Background:**

Accessing information that defines personally familiar context in real-world situations is essential for the social interactions and the independent functioning of an individual. Personal familiarity is associated with the availability of semantic and episodic information as well as the emotional meaningfulness surrounding a stimulus. These features are known to be associated with neural activity in distinct brain regions across different stimulus conditions (e.g., when perceiving faces, voices, places, objects), which may reflect a shared neural basis. Although perceiving context-rich personal familiarity may appear unchanged in aging on the behavioral level, it has not yet been studied whether this can be supported by neuroimaging data.

**Methodology/Principal Findings:**

We used functional magnetic resonance imaging to investigate the neural network associated with personal familiarity during the perception of personally familiar faces and places. Twelve young and twelve elderly cognitively healthy subjects participated in the study. Both age groups showed a similar activation pattern underlying personal familiarity, predominantly in anterior cingulate and posterior cingulate cortices, irrespective of the stimulus type. The young subjects, but not the elderly subjects demonstrated an additional anterior cingulate deactivation when perceiving unfamiliar stimuli.

**Conclusions/Significance:**

Although we found evidence for an age-dependent reduction in frontal cortical deactivation, our data show that there is a stimulus-independent neural network associated with personal familiarity of faces and places, which is less susceptible to aging-related changes.

## Introduction

When we perceive a personally familiar individual the identification process is modulated by person knowledge (e.g. personal traits, attitudes, and biographical facts), emotion, social attachment, and only in part based on visual facial appearance [1]. Functional magnetic resonance imaging (fMRI) studies investigating face familiarity show characteristic brain activation patterns associated with different cue types such as experimentally learned faces [2], famous person faces [3], or personally familiar faces [4]. Cloutier et al. [5] highlight that in addition to the well documented neural networks involved in face perception [6] familiarity effects can be divided into two major categories: *perceptually based familiarity* - faces a subject was previously exposed to but for which no surrounding information can be accessed, and *knowledge-based familiarity* when such person-associated knowledge is available.

In fMRI studies, perceiving experimentally learned faces activates the precuneus, supporting its role in familiarity processing, long-term memory retrieval and imagery [2], [7]. Famous person faces and personally familiar faces share the availability of background information to form the experience of recognizing a familiar individual. However, perceiving personally familiar faces when contrasted with famous faces is associated with greater anterior and posterior cingulate activation as well as greater activation in other brain regions [4]. This could be due to the richness of available episodic and semantic information associated with personal familiarity and might also reflect social attachment and emotional response [4], [8].

It has been hypothesized that semantic knowledge about a person's intentions or attitudes as well as emotional response will be activated spontaneously when we perceive a personally familiar face [4]. The regions involved could be part of a modular network that underlies person representation [9]. Thus, there is evidence that different types of person-specific semantic information, retrieval of related autobiographical episodes and emotional responses are associated with activity in distinct brain areas [1], [8]. Sugiura et al. [8] illustrate that due to the suggested modular nature of multimodal representation, different real-world representation processes may share distinct ‘modules’ or ‘cognitive response patterns’. This has been demonstrated for the neural network underlying personal familiarity that is consistently associated with activity in posterior cingulate/retrosplenial cortices for personally familiar names [8], places/objects [10] and faces/voices [11]. Although most studies aim at face perception, Epstein et al. [12] for example presented photographs of familiar and unfamiliar places during an fMRI experiment. The authors demonstrated that the retrospenial cortex showed the greatest signal increase within the network involved in scene perception when personally familiar locations were presented [12]. Additional anterior paracingulate and posterior superior temporal activations have been reported for personally familiar faces [4]. These areas are known to be associated with the representation of the mental states of others [13], self-referential processing [14], and the retrieval of autobiographical memories [15].

It has been consistently described in the literature that in contrast to recollection processes, familiarity processes are relatively unaffected by aging [16], [17], [18]. However, most studies refer to experimentally learned ( = familiar) stimuli. To our knowledge it has not yet been investigated whether normal aging affects the neural network associated with *personal* familiarity. This is particularly interesting because of the high social relevance of identifying personal familiar context throughout the lifespan. Although personally familiar stimuli and experimentally familiar stimuli share the fact that they are perceptually familiar, personally familiar stimuli are additionally associated with the spontaneous retrieval of rich background information and greater emotional response. Cloutier et al. [5] highlight that the availability of surrounding background information is associated with increased brain activity in medial prefrontal/anterior cingulate regions.

Perceptual familiarity processes remain stable in aging, whereas the age-associated decline in memory recollection performance and forming associations [19] could be due to altered neural functioning particularly in the prefrontal cortex [20]. Using positron emission tomography (PET) and different face processing experiments, Grady et al. [21] found a greater prefrontal neural response in older adults than in younger people. There is still a debate whether greater prefrontal brain activity would reflect neuronal dedifferentiation [21] or a compensatory mechanism [22]. Although this could lead to the idea that we may see age-related differences in the neural network associated with personal familiarity, the specific components defining the personal familiar experience were shown to be highly preserved in aging: access to semantic information [23], [24], retrieval of personally highly relevant episodic context [25], and emotional processing [26]. Furthermore, the ability to identify personally familiar information is essential for daily functioning and creating autobiographical context, which is supported by the overlapping neural networks underlying personal familiarity and autobiographical memory processing [1], [15], [27]. Finally, a similar neural network preferentially corresponding to personal familiarity can be found across different stimulus entities [1], [8], [10], [11]. This further strengthens its unique and preserved role in real-world representation processes.

We therefore predict that the neural network associated with personal familiarity remains preserved in the elderly when compared to young participants. Using fMRI we investigated the neural responses associated with the perception of personally familiar faces (close family members: e.g., spouse, children) and places (of the subjects' own home) when contrasted to unfamiliar faces and places in cognitively healthy young and elderly individuals.

## Methods

### Subjects

All subjects responded to public advertisements. The University's Ethics Committee for Medical Research (Ethics Committee of Dresden University's Medical Faculty ‘Carl Gustav Carus’, Dresden, Germany) approved the study, and written informed consent was obtained. Twelve young and twelve elderly subjects participated in the study. All participants were cognitively healthy. All participants were right-handed, underwent medical history evaluation, neuropsychological testing, and structural brain MRI. Only subjects free of white matter lesions or focal white matter lesions only (score <2 points, ARWMC-scale, [28]), and free of focal lesions in grey matter were included. Exclusion criteria were an education of less than eight years, a history of alcohol or substance abuse, head trauma, a psychiatric or neurological disorder, or major systemic disease affecting brain function. Sociodemographic data and neuropsychological scores were compared using two-tailed t-tests (for details see **Table 1**).

**Table 1:** Demographic and clinical characteristics.

Characteristic (range), mean±SD	Young subjects(N = 12)	Elderly subjects(N = 12)
Age (years)	30.4±6.1	62.1±5.4
School education (years)	12.0±0.0	11.1±1.4
Female sex (no.)	6	6
MMSE (0–30)	30.0±0.0	29.6±0.5
CDR (0–3)	0.0±0.0	0.0±0.0
WMS-R		
- digit span forward (0–12)	9.4±1.6	9.4±1.6
- digit span reverse (0–12)	7.9±2.3	8.4±2.0
- visual memory, copy (0–41)	39.4±1.8	38.5±3.0
- visual memory, delayed (0–41)	36.9±3.4	36.7±4.4
COWAT (no.)	50.3±11.5	48.8±12.7
Trail Making Test, A (sec.)	22.5±5.1	37.1±14.0*
Trail Making Test, B (sec.)	58.6±15.2	84.8±48.7*
CVLT		
- short delay free recall (0–16)	13.2±1.6	11.3±3.3
- short delay cued recall (0–16)	13.6±1.2	12.1±2.6
- long delay free recall (0–16)	13.7±1.2	11.5±3.3*
- long delay cued recall (0–16)	13.5±1.2	12.5±2.3
- recognition hits (0–16)	15.4±0.7	14.7±1.2
- false positive (0–28)	0.25±0.6	1.7±2.1*
- List B (0–16)	7.2±2.3	5.6±2.4

MMSE, Mini Mental State Examination; CDR, Clinical Dementia Rating; WMS-R,

Wechsler Memory Scale – Revised; COWAT, Controlled Oral Word Association Test; CVLT, California Verbal Learning Test;

*p<0.05, 2-tailed.

### Stimuli

For the familiar faces, pictures of each participant's close relatives (e.g., spouse, children) were taken with a digital camera. All the relatives were photographed from 5 different angles (left side; 45° left, frontal, 45° right, right side). The images were digitally manipulated to ensure similar head size, luminance, and background. Pictures of unfamiliar faces were taken of family members of the clinical staff using the same angles as for the close relatives. Familiar and unfamiliar face stimuli were matched for gender and approximate age. Images of familiar places were taken of the participants' homes. We obtained photographs of complete rooms rather than of individual pieces of furniture. Pictures of unfamiliar places were obtained from the homes of clinical staff members and their relatives.

**Figure 1:**
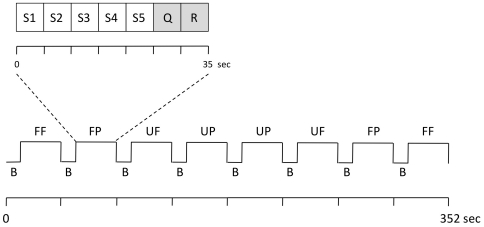
fMRI paradigm. This figure visualizes one experimental run of the fMRI paradigm. Three of these runs, each lasting 352 s, were performed. The order of the four conditions (FF, UF, FP, UP) was counterbalanced across the runs. Each block (35 s) of a condition consisted of a visual stimulus presented from five different angles (S1-5), a familiarity question (Q), and a response (“thank you”, R). FF = familiar face, UF = unfamiliar face, FP = familiar place, UP = unfamiliar place, B = baseline (fixation cross, 9 s).

According to our experimental design (see below and **Figure 1**) each subject was presented six familiar and six unfamiliar faces as well as six familiar and six unfamiliar places. Within each condition, a stimulus was presented five times. To avoid habituation effects the stimulus image was presented from five different angles and an individual stimulus image was not repeated within or across the experimental conditions. Therefore we utilized a total of 120 images (60 images for faces and 60 images for places) for each study subject.

### Experimental design

Utilizing a blocked factorial design, the subjects were presented images of personally familiar faces and places, as well as unfamiliar faces and places during the fMRI experiment to evoke the neural responses associated with different stimulus modality and familiarity. Five individual stimuli of one of the four conditions were blocked together (stimulus onset-time 5 s). The images were presented in counterbalanced order within and between the three runs for both familiarity and stimulus modality. The order of stimulus presentation did not vary across subjects. To control for alertness and to test whether participants would correctly recognize familiar and unfamiliar stimuli each block contained a question stimulus (“if the stimulus presented is familiar, press the button in your left hand/if unfamiliar, press the button in your right hand”) in response to which the subjects pressed a button on a keypad in their right or left hand. This question (duration 5 s) was presented after the visual stimuli and followed by a “thank you” response (duration 5 s). Experimental conditions were separated by intervals lasting 9 s, during which the participants focused on a fixation cross (for experimental design see also **Figure 1**). Using a 3T MRI scanner (Trio; Siemens AG, Erlangen, Germany), we performed a total of three experimental runs, each consisting of 8 stimulus blocks. fMRI images were acquired with an EPI pulse sequence using BOLD contrast: TR = 1.95 s, TE = 25 ms, α = 80°, 34 transversal slices in descending order, orientated axially parallel to the ac-pc line, thickness 3 mm (1 mm gap), FOV = 220 mm, voxel size 3.44×3.44×4 mm^3^. We collected 547 volumes for each subject. Stimuli were presented using bi-screen goggles, placed next to the subject's eyes below the head coil (VisuaStim Digital, Resonance Technology Inc., Northridge, CA, USA). The task presentation and the behavioral response recording were performed with Presentation® software (Version 9.9, Neurobehavioral Systems Inc., Albany, CA, USA). Prior to functional neuroimaging, high-resolution anatomic images were acquired using a T1-weighted 3-D magnetization-prepared, rapid acquisition gradient echo (MPRAGE) pulse sequence: TR = 1.9 s, TE = 2.26 ms, FOV = 256 mm, 176 slices, voxel size 1×1×1 mm^3^.

### Image processing and statistical analysis

Image processing and statistical calculations were performed using MATLAB (The Mathworks Inc., Natick, MA, USA) and statistical parametric mapping software (SPM5, Wellcome Department of Imaging Neuroscience, London, UK). The first five EPI images were discarded to allow the MRI signal to reach a steady state. Individual data were spatially realigned to correct for head movement. For normalization we used a standard EPI template (MNI brain). After resampling to achieve 3×3×3 mm isotropic voxels the functional data were smoothed using an isotropic Gaussian kernel of 10 mm FWHM. On the single subject level, we modelled all four conditions of the paradigm (familiar faces, unfamiliar faces, familiar places, unfamiliar places) in the context of the general linear model (GLM). We also modelled the question stimulus and the subjects' response (button presses) separately from the rest condition (focusing on a fixation cross). We used a flexible factorial modelling procedure for second level analyses in a 2*2*2 factorial design, investigating the factors stimulus type (face/place), familiarity (familiar/unfamiliar), and group (old/young). We were specifically interested in the effect associated with personal familiarity irrespective of stimulus type on the neural activation patterns within and between groups (FF+FP>UF+UP and UF+UP>FF+FP; FF = familiar faces, UF = unfamiliar faces, FP = familiar places, UP = unfamiliar places). We further examined additional interaction effects between stimulus type and familiarity [(FF>UF)>(FP>UP), (FP>UP)>(FF>UF), (UF>FF)>(UP>FP), (UP>FP)>(UF>FF)] within and between groups. Voxels in MNI-space were considered statistically significant at a threshold of p<0.05 (corrected at cluster level) using a height threshold of p<0.001 uncorrected, corresponding to T = 3.28. To investigate possible activation changes in the anterior cingulate and posterior cingulate cortex, a region of interest (ROI) approach was chosen based on the coordinates of Sugiura et al. [29]: −6, 36, −2 for the anterior cingulate cortex and −2, −42, 40 for the posterior cingulate cortex, applying small volume correction using a sphere centered at these coordinates with a radius of 10 mm corresponding to the size of the Gaussian kernel used for smoothing.

## Results

### Neuropsychological test results

Subjects performed within the normal range (age adjusted z-values +/−1z, not shown) in all neuropsychological tests. However, when compared to elderly participants, the younger group performed significantly better in tests addressing speed of information processing, delayed word list recall, and word list recognition intrusions (for details see **Table 1**). Using these data as additional regressors in the fMRI analysis did not change the results. There was no significant group difference in the reaction times in the subordinate alertness task during the fMRI experiment. All the subjects correctly assigned all the stimuli to the ‘familiar’ or the ‘unfamiliar’ category.

### fMRI

**Figure 2:**
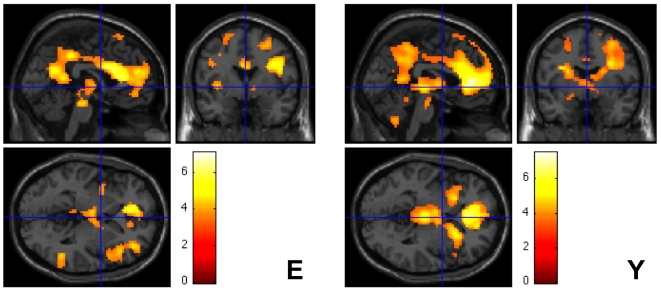
Effect of personal familiarity irrespective of stimulus type. The figure shows brain areas with a relative increase in neural activity associated with familiar>unfamiliar stimulus content in elderly (E) and young (Y) participants irrespective of stimulus type. Given are SPM maps showing Z-values (color scale) superimposed on a SPM5 standard single subject's brain sections. The coordinates of local maxima and the corresponding Z-values are given in Table 2. The activations shown are significant at p<0.05, corrected for multiple comparisons at the cluster level. The group comparison between elderly and young subjects revealed no statistically significant differences.

**Figure 3:**
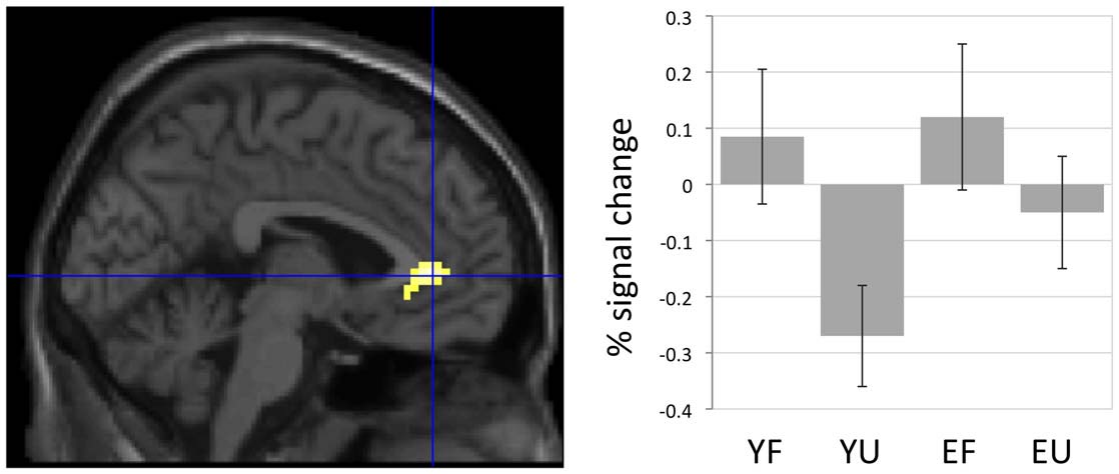
Interaction between age and familiarity. The figure shows an area (anterior cingulate cortex, −3, 39, 3) in which the young but not the elderly subjects showed reduced neural activity for unfamiliar versus familiar stimuli irrespective of stimulus type. Signal change at the local maximum is statistically significant at the voxel level (Psvc<0.05) in a ROI analysis based on the coordinates by [29]. The local maximum is superimposed on a sagittal single subject brain section provided by SPM5. The histogram displays percentage BOLD signal change for the local maximum as a function of the experimental conditions (mean and 90% confidence interval). YF = young familiar, YU = young unfamiliar, EF = elderly familiar, EU = elderly unfamiliar.

Familiar compared to unfamiliar stimuli, irrespective of stimulus type (FF+FP>UF+UP), elicited substantially more brain activity, which was primarily located in anterior cingulate and posterior cingulate areas (**Table 2**, **Figure 2**). The young and the elderly subjects showed a similar activation pattern. For whole brain analysis, group comparison did not yield any effects that reached statistical significance. In a hypothesis-driven ROI analysis the younger subjects showed a significantly greater BOLD signal change in the anterior cingulate cortex in comparison to the elderly subjects. This was due to decreased activity in this region for unfamiliar versus familiar stimuli among the young subjects. The elderly subjects did not show this regional deactivation (**Table 2**, **Figure 3**). The analysis of other interaction terms between the factors stimulus type and familiarity [(FF>UF)>(FP>UP), (FP>UP)>(FF>UF), (UF>FF)>(UP>FP), (UP>FP)>(UF>FF)] did not reveal significant results within or between groups.

**Table 2:** Relative increases in brain activity associated with personal familiarity.

Region	Side	x	y	z	T(Z)-Score	k_E_ (voxels)
Main effect of familiar versus unfamiliar: (FF + FP) > (UF + UP)						
**Elderly subjects**						
Anterior cingulate	L	−9	45	17	7.11(5.79)	5553#
Posterior cingulate	L	−12	−51	30	6.67(5.53)	#
Middle frontal gyrus	L	−24	30	39	4.67(4.2)	102
Inferior frontal gyrus	L	−30	24	−18	5.07(4.49)	133
Inferior frontal gyrus	L	−51	6	12	4.62(4.19)	174
Precentral gyrus	L	−33	−12	51	4.62(4.16)	219
Inferior parietal lobule	L	−36	−42	45	4.25(3.87)	108
Cerebellum	R	33	−54	−51	5.58(4.84)	130
Inferior temporal gyrus	R	51	−51	3	5.17(4.55)	151
**Young subjects**						
Anterior cingulate	L	−3	39	6	7.52(6.02)	7512#
Posterior cingulate	L	−3	−51	33	4.62(4.16)	#
Inferior frontal gyrus	L	−45	−60	24	4.85(4.33)	373
Precentral gyrus	L	−15	−45	−48	5.43(4.73)	710
Middle temporal gyrus	L	−48	−30	−18	4.5(4.07)	175
Cerebellum	L	−33	39	9	5(4.43)	128
Inferior temporal gyrus	R	60	−54	−9	4.93(4.38)	134
**Elderly > Young subjects**						
Anterior cingulate	L	−3	39	3	3.84(3.68)	34*

All activations are significant at p<0.05, corrected for multiple comparisons at the cluster level (with a height threshold of p<0.001, uncorrected at the voxel level).

* = p_SVC_<0.05 in a hypothesis-driven region-of-interest (ROI) analysis. For each region of activation, the coordinates of the maximally activated voxels within the activation cluster are given in standard stereotactic MNI space. FF: familiar faces, UF: unfamiliar faces, FP: familiar places, UP: unfamiliar places;

# indicates that this activation maximum is part of the same cluster.

## Discussion

The aim of this study was to investigate whether aging affects the neural networks underlying personal familiarity when perceiving personally familiar versus unfamiliar faces and places. We revealed that the general neural network associated with personal familiarity did not differ between the young and the elderly people. Both age groups activated frontal and anterior cingulate as well as posterior cingulate and temporal cortices irrespective of stimulus type (face/place). In a hypothesis-driven region of interest analysis we further demonstrated that compared to the elderly participants, the young subjects showed a greater neural deactivation in the anterior cingulate cortex when perceiving unfamiliar versus familiar stimuli. This effect was also stimulus type-independent.

Elderly study participants had an average age of 62±5 years to ensure age comparability with people experiencing very early pathologic cognitive decline in subsequent studies. Therefore our data should be interpreted with respect to this age group.

First, our data are in line with previous observations that within a modular structure of neural networks involved in real-world representation, brain activity associated with personal familiarity shows a relatively preserved pattern across various stimuli such as faces, voices, places, and objects [1], [8], [10], [11]. Furthermore, we contribute to the existing evidence that multimodal representation processes involve neural networks that are differentially affected by aging. Previous studies showed age-related changes in neural activation patterns associated with recognizing visual stimuli [21] or mental visualization [30]. In contrast, the neural networks associated with perceiving personal familiarity may remain stable in the aging brain. Various studies primarily aimed at face processing demonstrated key elements involved in creating a personally familiar context: availability of person-specific semantic information, retrieval of related autobiographical episodes and emotional responses [8], [9], [31]. These components are highly preserved in aging [23], [24], [25], [26]. Older people tend to rely on semantic information to aid autobiographical memory retrieval [23]. Whereas specific event details become less accessible in aging, the preservation of semantic memories is fundamental for personal identity [23], [32]. Furthermore, self-defining memories are less likely to be affected by aging, suggesting that the degree of self-relevance of the familiar stimulus is associated with information accessibility [25], [32]. Older adults were shown to remember the affective details of an event better than the non-affective details of an event [26]. There is evidence that elderly people may show better performance in processing positive emotional responses, especially in respect to face stimuli [33]. However, Kensinger [26] suggests that memories for affective context may rely on relatively preserved neural networks in older individuals.

It is important to be aware of the different context in which familiarity was investigated. This study as well as investigations by Sugiura et al. [10] or Gobbini et al. [4] primarily aimed at the perception and the identification of personally familiar versus unfamiliar stimuli. Others focused on the recognition of previously learned (familiar) versus unlearned (unfamiliar) material in the context of episodic memory performance [19], [34]. In that context familiarity is thought to be a signal detection process whereby items are categorized as having been studied or not [34] while no specific associations or contextual details are retrieved. In contrast, recollection refers to memories of past events that include contextual details and specific associations. Both processes contributing to memory retrieval are associated with different neural networks [35] and are differentially susceptible to aging-related changes. Aging primarily affects recollection and integration of specific details rather than familiarity-associated recognition [17], [18]. Older people have been shown to depend on familiarity to compensate for recollection deficits [36], [37].

Personal familiarity in contrast to experimentally learned familiarity differs on the level of available contextual knowledge and emotional response. Gobbini and Haxby [1] highlight that neuroimaging studies on familiar face perception may have shown inconsistent results due to the different types of familiarity that were investigated (experimentally learned faces, familiarity associated with famous faces, or personal familiarity). Recent data confirm that different neural networks are involved in perceptual familiarity and the availability of knowledge about the stimulus. Cloutier et al. [5] demonstrate that the posterior cingulate cortex/precuneus is involved in perceptual face familiarity. This is in line with investigations by others showing activity in this region associated with experimentally learned faces when compared to new faces [2]. However, the region's association with episodic memory [27] and familiarity of non-face stimuli [10], [11] supports a more complex role in the representation of familiar targets [5]. Medial prefrontal cortical activation may be associated with the availability of background knowledge for a stimulus [5]. The coordinates found by Cloutier et al. [5] are close to an anterior paracingulate activation described by Gobbini et al. [4] for perceiving personally familiar versus famous persons. This strengthens the theories about the region's role in social cognition, self-referential processing and the capacity to represent and interpret the mental state of others [5], [38], [39].

Previous studies investigating experimentally learned familiarity showed that this ‘perceptual’ familiarity is preserved in aging [16], [17], [18]. We have expanded these findings by demonstrating that the neural network underlying *personal* familiarity appears to be preserved as well. This may be due to the involvement of extensive semantic contributions and the processing of emotionally meaningful and self-relevant stimuli, areas of cognitive functioning in which elderly people generally show a good performance [23], [25], [26].

Our finding that older participants do not show an anterior cingulate deactivation for unfamiliar stimuli may also point into the direction of future familiarity/aging research. In a semantic classification task, Lustig et al. [40] showed reduced deactivation among cognitively healthy elderly people specifically in medial frontal and posterior cingulate regions. The authors additionally investigated demented subjects and found no further reduction in deactivation in the medial frontal cortex when compared to non-demented older people [40]. This suggests an age-related rather than a disease-associated effect for the reduced deactivation in the medial frontal cortex. The frontal brain coordinates showing peak activity reported by Lustig et al. [40] are close to the region where we found reduced deactivation for unfamiliar stimuli among elderly subjects. The functional relevance of reduced deactivations in aging is not yet well understood. Task difficulty for example, has been shown to be associated with a greater deactivation in specific brain areas among young subjects [41], [42]. A reduced deactivation magnitude in older adults might reflect a reduced sensitivity do task demand [42]. Miller et al. [43] suggest that an age-related decline in memory performance might be related to default mode activity suppression failure during memory formation in older adults. However, in this study we did not directly examine default mode network differences between younger and older adults. As an alternative hypothesis, it seems also possible that due to age-related impairment in inhibitory processes [44] older adults are less able to completely ‘inhibit’ the processing of familiar items when viewing unfamiliar stimuli. To test the hypothesis we additionally investigated brain activity during the baseline intervals following the familiar conditions. This analysis revealed no difference in brain activity between both groups. Moreover, within group, brain areas that have been shown to be preferentially associated with familiarity processing (e.g., precuneus/posterior cingulate cortex) or the availability of contextual information surrounding a familiar stimulus (e.g., medial prefrontal cortex/anterior cingulate cortex) did not show differential activity during the baseline intervals following the presentation of familiar stimuli. Together this suggests that independent of age all the participants were able to inhibit the processing of a familiar stimulus when the familiar stimulus was no longer present.

This study has several limitations. Our data are susceptible to false negative findings due to the relatively small subject sample size and the rather strict statistical threshold. We therefore reanalyzed our data applying a threshold of p<0.05, uncorrected, which did not change our findings. In this study, we only investigated high frequency personal familiarity. Future studies could determine how other personal familiar stimuli (e.g., more distant relatives) would modulate the pattern of activated brain regions. Due to the higher autobiographical or emotional relatedness, personally familiar stimuli, when compared to unfamiliar stimuli, could have been perceived in a more gestalt manner. If so, this could be a confound because in this case the block design may have preferentially boosted the effects elicited by familiar stimuli over those associated with the unfamiliar stimuli. Furthermore, compared to younger subjects, older subjects might have greater difficulties in inhibiting familiar stimuli processing when perceiving unfamiliar stimuli. Investigating brain activity during the baseline intervals following familiar stimuli did not suggest such an effect in our sample, even when applying the less stringent statistical threshold mentioned above. However, it is still possible that we may not have had enough statistical power to detect this effect. Although replicating our findings by using an event-related fMRI design might be of interest, our main finding of the neural network underlying personal familiarity being less susceptible to aging-related changes remains robust.
